# Multiple-bias analysis as a technique to address systematic error in measures of abortion-related mortality

**DOI:** 10.1186/s12963-016-0075-3

**Published:** 2016-03-22

**Authors:** Caitlin Gerdts, Jennifer Ahern

**Affiliations:** 1 Ibis Reproductive Health, 1330 Broadway St, Suite 1100, Oakland, CA 94612 USA; 2 Division of Epidemiology, University of California, Berkeley, School of Public Health, Berkeley, CA USA

## Abstract

**Background:**

The UN Millennium Development Goals (MDGs) and Sustainable Development Goals (SDGs) have brought heightened global attention to the measurement of maternal mortality. It is imperative that new and novel approaches be used to measure maternal mortality and to better understand existing data. In this paper we present one approach: an epidemiologic framework for identifying the identification and quantification of systematic error (multiple-bias analysis), outline the necessary steps for investigators interested in conducting multiple-bias analyses in their own data, and suggest approaches for reporting such analyses in the literature.

**Methods:**

To conceptualize the systematic error present in studies of abortion-related deaths, we propose a bias framework. We posit that selection bias and misclassification are present in both verbal autopsy studies and facility-based studies. The multiple-bias analysis framework provides a relatively simple, quantitative strategy for assessing systematic error and resulting bias in any epidemiologic study.

**Results:**

In our worked example of multiple-bias analysis on a study reporting 20.6 % of maternal deaths to be abortion related, after adjustment for selection bias, misclassification, and random error, the median increased, on average, to 0.308, approximately 20 % greater than the reported proportion of abortion-related deaths.

**Conclusions:**

Reporting results of multiple-bias analyses in estimates of abortion-related mortality, predictors of unsafe abortion, and other reproductive health questions that suffer from similar biases would not only improve reporting practices in the field, but might also provide a more accurate understanding of the range of potential impact of policies and programs that target the underlying causes of unsafe abortion and abortion-related mortality.

**Electronic supplementary material:**

The online version of this article (doi:10.1186/s12963-016-0075-3) contains supplementary material, which is available to authorized users.

## Background

The launch of the UN Millennium Development Goals (MDGs) in 2000 brought heightened global attention to the importance of reducing levels of maternal mortality [1]. The new Sustainable Development Goals (SDGs) build on the momentum established by the MDGs [2], and Sustainable Development Goal 3.1 calls for a reduction of the global maternal mortality ratio to less than 70 per 100,000 live births [3]. In order to document such a global reduction, however, all countries must have the capacity to accurately measure maternal deaths, a task which has proven to be a decades-long challenge [4–6]. Weak infrastructure, lack of functional civil vital registration systems, and misclassification of maternal deaths have posed significant obstacles to the accurate measurement of maternal mortality in much of the developing world [7, 8].

Despite problems with data quality, existing evidence suggests that abortion-related policies may play a key role in maternal mortality reduction globally. Since the MDGs were established, precipitous nationwide reductions in maternal mortality have occurred following the liberalization of abortion laws in Nepal and South Africa [9, 10]. It is plausible that in countries where abortion is made legal and available, not only is maternal mortality due to unsafe abortion greatly reduced, but governments may be able to focus more effectively on other causes of maternal mortality, thereby leading to faster reductions in overall maternal mortality compared to countries where unsafe abortion is a major cause of maternal death.

In order to better understand existing maternal mortality data, and improve the measurement of cause-specific maternal mortality, novel approaches to data analysis, reporting, and collection must be tested. Here we present a simple, theoretical framework for identifying bias in reproductive health studies (specifically as applied to estimates of abortion-related mortality), outline the steps for the quantification of bias in such studies, and suggest approaches for reporting such analyses in the literature.

## Methods

### Bias framework for studies of abortion and maternal mortality

**Fig. 1:**
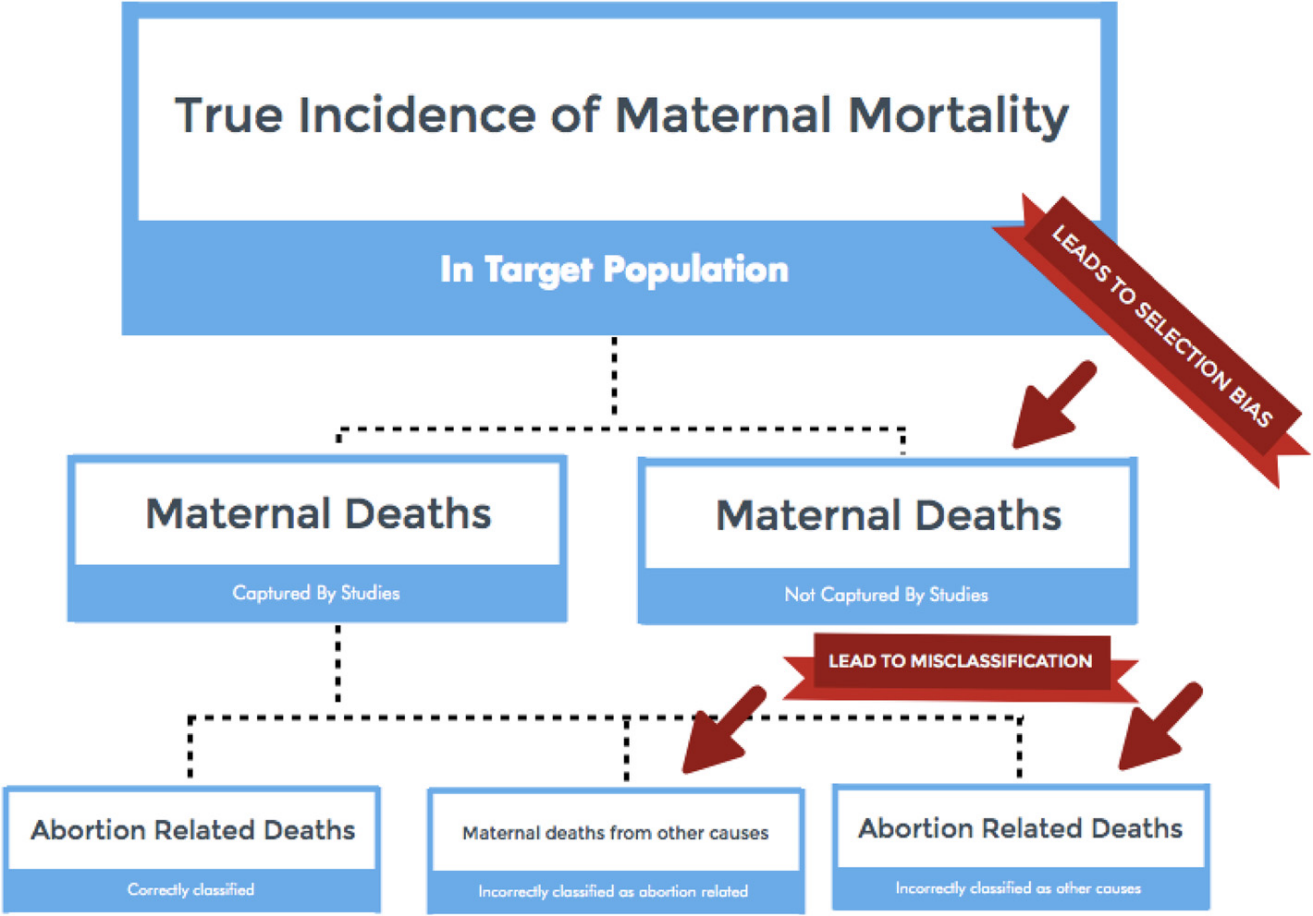
Bias Framework

Abortion-related mortality is uniquely prone to bias for a number of reasons: 1) in countries where abortion is restricted or illegal altogether, women often seek abortion-related services outside of the formal medical system; 2) in such settings, due to social and cultural stigma, and fear of legal consequences, women are often reluctant to seek medical services in the event of complications or reveal to family members the underlying cause of the complications [11–18]; and 3) because of legal consequences for patients and providers alike, clinicians who provide abortion-related services may be reluctant to report abortion-related complications or deaths [12, 19, 20]. To conceptualize the systematic error present in studies of abortion-related deaths, we propose a bias framework (Fig. 1). We posit that two types of bias are present in all studies of abortion-related mortality: 1) selection bias and 2) misclassification.

Selection bias arises when the study population is systematically different from the target population with respect to exposure or outcome. In the case of abortion-related mortality due to legal, social, and economic obstacles to safe abortion care, women who experience abortion-related deaths are less likely to come to health facilities relative to women who do not experience abortion-related death, and, because of the stigma surrounding abortion, are also less likely to have family members who can reliably participate in verbal autopsy studies of maternal deaths. Therefore, selection bias is likely to bias study results (i.e., the number of maternal deaths attributed to abortion that are measured in a study will differ from the number of maternal deaths actually attributable to abortion within the target population) [21].

Misclassification arises when measurement of study variables is flawed, resulting in a study subject being incorrectly classified with respect to the outcome (or exposure) of interest. In the case of abortion-related mortality, women who experience abortion-related deaths are more likely to have their deaths be incorrectly classified as other causes of maternal death than women who do not experience abortion-related deaths. Misclassification is, therefore, likely to bias study results (i.e., the sensitivity and specificity of abortion-related classification will differ from the sensitivity and specificity for deaths from other maternal causes, and the proportion of maternal deaths due to abortion in the study will differ from the proportion in the study population) [21].

### Epidemiologic approaches to quantify systematic error

Techniques for the quantitative assessment of systematic error have existed for decades [22], and range from simple sensitivity analyses [23] to complex Bayesian uncertainty analyses [24]. Yet, it is only recently that calls have emerged in the epidemiologic literature to evaluate and report levels of systematic error [24–26]. One suggested technique is multiple-bias analysis, a probabilistic extension of basic sensitivity analysis that allows investigators to address multiple non-independent threats to a study’s validity in one analysis (e.g., selection and misclassification bias, simultaneously) [24]. Multiple-bias analysis requires three main components: first, researchers determine which biases (for example, information bias and/or selection bias) are likely to exist in their studies. Second, using expert knowledge and data from validation studies (where these studies exist), researchers construct parameters (or distributions of parameters) of the probable magnitude of those biases. Third, after applying the distributions (“bias parameters”) to the data, researchers randomly sample from those parameters thousands of iterations to generate hypothetical distributions of point estimates, had the postulated biases not existed in the study.

### A multiple-bias analysis plan

To adjust the results of any study of abortion-related mortality using multiple-bias analysis techniques, eight straightforward steps can be implemented. Step 1: Specify probability distributions for the selection probabilities of abortion-related deaths and non-abortion-related deaths in each study. Step 2: Specify probability distributions for the sensitivity and specificity of classifying abortion-related deaths for each study. Step 3: Using crude data from each study of interest, calculate the proportion of abortion-related deaths in the study. Step 4: Construct 95 % confidence intervals for the reported proportion of abortion-related deaths for the study. Step 5: Adjust the reported proportion of abortion-related deaths for selection bias in the study. Step 6: Using the selection-bias adjusted proportion of abortion-related deaths, subsequently adjust for misclassification in the study. Step 7: Incorporate random error into the adjusted estimates for the study of interest and construct a range of possible values for the proportion of abortion-related deaths adjusted for selection bias, misclassification, and random error. Step 8: Model an a priori established quantity of Monte Carlo simulation trials for each simulation experiment under different probability distribution scenarios.

### Detailed steps and mathematical formulae for conducting multiple-bias analyses of abortion-related mortality

Step 1: Specify the shape and width of probability distributions for the selection probabilities of abortion-related deaths and non-abortion-related deaths (i.e., the probability that abortion-related deaths that should be captured by a study are, in fact, enumerated by that study). Under ideal circumstances, selection probabilities would be determined via internal validation studies; however, given that few internal validation studies exist in the literature, selection probabilities should be approximated using 1) data from validation studies of maternal mortality conducted in similar regions/populations and 2) adjustment factors commonly used in the demographic literature to adjust for underestimation of maternal death in studies of maternal mortality and abortion-related mortality [27]. While these sources of probability distributions are imperfect proxies for the real selection probabilities, by constructing probability distributions of a range of possible values of selection bias, we can explicitly state the range of selection bias that one assumes, and model what the data would have looked like given a random sampling of those possible values. Trapezoidal distributions are the most commonly employed shape for probability distributions in the multiple-bias analysis literature [24, 25, 28] as they allow for the specification of the range of most likely values (between the lower and higher modes of the trapezoid) and the range of all possible values (between minimum and maximum specified values).

Step 2: Specify shape and width of probability distributions for the sensitivity and specificity of classifying abortion-related deaths for each study. Specify probability distributions for the sensitivity and specificity of classifying abortion-related deaths for each study. As in Step 1, two sources of information should be used to specify these probability distributions: 1) data from validation studies of verbal autopsy algorithms conducted in the same country or in similar populations, and 2) data from validation studies conducted in the same country (or in similar populations) of cause of death classification from clinical case notes against autopsy diagnoses. While these two sources of data are imperfect, there is substantial validation literature testing the sensitivity and specificity of cause of death classification in different parts of the world that was used to inform our choices of bounds for the range of possible values of sensitivity and specificity [29–33].

Step 3: Using crude data from each study of interest, calculate the proportion of abortion-related deaths in each study with the following formula:

1 $$ {\mathbf{Y}}_{\mathbf{0}}=\frac{X_{0 ARD}}{Tota{l}_{MD}}, $$

where Y_0_ is the proportion of observed the number of maternal deaths abortion-related deaths (ARD), X_*0ARD*_ is the number of abortion-related deaths identified by the study, and *Total*_*MD*_ is the total number of maternal deaths identified by the study of interest.

Step 4: Construct a probability distribution and a 95 % confidence interval of the proportion calculated in Step 3 using the following formulae:

1. *SE = sqrt(Y*_0_*(1-Y_*0*_*)/(Total*_*MD*_*)),* where *SE* is the standard error of *Y*_*0*_, and *Y*_*0*_ is the proportion observed abortion-related deaths in the study of interest.
2. *95 % CI = Y*_*0*_ ± SE

Step 5: Adjust for selection bias in the study using the following formulae:

1. ${\mathbf{X}}_{\mathbf{1ARD}}=\left(\frac{X_{0 ARD}}{W_1}\right)$ where *X*_*1ARD*_ is the number of abortion-related deaths adjusted for selection bias, *X*_*0ARD*_ is the number of abortion-related deaths identified by the study of interest, and where *W*_*1*_ is the a priori specified trapezoidal distribution of all possible values for the selection probability for abortion-related deaths.
2. ${\mathbf{X}}_{\mathbf{1NARD}}=\left(\frac{X_{0 NARD}}{W_2}\right)$ where *X*_*1NARD*_ is the number of non-abortion-related maternal deaths adjusted for selection bias*, X*_*0NARD*_ is the number of non-abortion-related maternal deaths identified by the study of interest, and where *W*_*2*_ is the selection probability for non-abortion-related maternal deaths.
3. ${\mathbf{Y}}_{\mathbf{1}}=\frac{\left(\frac{X_{0 ARD}}{W_1}\right)}{\left(\frac{X_{0 ARD}}{W_1}+\frac{{X_0}_{NARD}}{W_2}\right)}$ where Y_1_ is the proportion of abortion-related deaths observed in the study of interest adjusted for selection bias, and other notation is as above.

It should be noted that the proportion of abortion-related deaths should be adjusted in the order in which the biases occurred. Given that subjects must, by necessity, be selected into any study before misclassification can occur, adjustment for selection bias comes first, then misclassification bias.

Step 6: Adjust for misclassification in the study of interest. Given that misclassification can only occur among subjects selected into any study, *Y*_*1*_ (the proportion of abortion-related deaths observed in the study adjusted for selection bias) should be utilized as the baseline for misclassification adjustment via the following formulae:

1. **X**_**2ARD**_ = [(*X*_1*ARD*_ * *W*_3_) + (*X*_1*NARD*_ − (*X*_1NARD_*W_4_))] where *X*_*2ARD*_ is the number of abortion-related deaths adjusted for selection bias and misclassification, *X*_*1ARD*_ is the number of abortion-related deaths adjusted for selection bias, *X*_*1NARD*_ is the number of non-abortion-related maternal deaths adjusted for selection bias, and where W_3_ is the sensitivity of classification of abortion-related death and *W*_*4*_ is the specificity of classification of abortion-related death.
2. **X**_**2NARD**_ = [(*X*_1*NARD*_ * *W*_3_) + (*X*_1*ARD*_ − (*X*_1*ARD*_ * *W*_4_)] where *X*_*2ARD*_ is the number of non-abortion-related deaths adjusted for selection bias and misclassification, *X*_*1ARD*_ is the number of abortion-related deaths adjusted for selection bias, *X*_*1NARD*_ is the number of non-abortion-related maternal deaths adjusted for selection bias, and where W_3_ is the sensitivity of classification of abortion-related death and *W*_*4*_ is the specificity of classification of abortion-related death.
3. ${\mathbf{Y}}_{\mathbf{2}}=\frac{X_{2 ARD}}{X_{2 ARD}+{X}_{2 NARD}}$ where *Y*_*2*_ is the proportion of abortion-related deaths adjusted for selection bias and misclassification, *X*_*2ARD*_ is the number of abortion-related deaths adjusted for selection bias and misclassification, and *X*_*2NARD*_ is the number of non-abortion-related maternal deaths adjusted for selection bias and misclassification.

Step 7: After adjusting for both sources of bias (selection bias and misclassification) incorporate random error into the new estimate. Using the same formulae that were employed in Step 1, construct a probability distribution and a range of possible values for the proportion:

1. *SE = sqrt(Y*_2_*(1-Y_*2*_*)/(Total*_*MD*_*)),* where *SE* is the standard error of *Y*_*2*_ and *Y*_*2*_ is the proportion of observed abortion-related deaths in the study of interest, adjusted for selection bias and misclassification.
2. *95 % CI = Y*_*2*_ ± SE.

Step 8: Model 50,000 Monte Carlo simulation trials for each simulation experiment under different probability distribution scenarios, with 21 scenarios in total.

## Results

### A worked example of multiple-bias analysis

In order to test a working example of multiple-bias analysis and evaluate the influence of selection bias and misclassification in a study of abortion-related maternal mortality, we use a published study of maternal deaths from the maternity ward of a main referral hospital in a major urban center in East Africa over a seven-year period [34]. This study was selected at random from the studies included in a systematic review of abortion-related mortality literature [35]. For the purposes of this worked example, let us refer to the selected study as “Study A.” Study A identified 253 maternal deaths, of which 52 were abortion-related.

In this example, we applied a multiple-bias analysis framework to estimates of abortion-related mortality and performed multiple-bias analyses on estimates of the proportion of abortion-related mortality reported by Study A. We hypothesized that both selection bias and misclassification were present in Study A. We generated prior probability distributions for selection bias and misclassification from existing external validation studies [36–40] and commonly employed demographic adjustment factors [27]. We developed and tested 21 different bias parameter scenarios for Study A, exploring all possible combinations of the 21 prior probability distributions in our analyses to identify trends in the generated bias-adjusted estimates for abortion-related mortality. The specific values of the parameters employed for this analysis are presented in Table 1, and the statistical code using the R statistical software package [41] is provided in The Additional file 1.

**Table 1:** Descriptions of trapezoidal probability distributions used for multiple-bias analysis of Study A

Study A	Scenario	W_1_^a^	W_2_^a^	W_3_^a^	W_4_^a^	RE
	1	0.2, 0.34, 0.43, 0.78	0.5, 0.7, 0.8, 1.0			None
	2	0.2, 0.30, 0.50, 0.78	0.5, 0.65, 0.85, 1.0			None
	3	0.2, 0.24, 0.53, 0.78	0.5, 0.6, 0.9, 1.0			None
	4	0.2, 0.34, 0.43, 0.78	0.5, 0.7, 0.8, 1.0	0.6, 0.7, 0.8, 0.9	0.91, 0.95, 0.97, 0.99	None
	5	0.2, 0.34, 0.43, 0.78	0.5, 0.7, 0.8, 1.0	0.6, 0.65, 0.85, 0.9	0.91, 0.94, 0.98, 0.99	None
	6	0.2, 0.34, 0.43, 0.78	0.5, 0.7, 0.8, 1.0	0.6, 0.62, 0.82, 0.9	0.91, 0.92, 0.98, 0.99	None
	7	0.2, 0.30, 0.50, 0.78	0.5, 0.65, 0.85, 1.0	0.6, 0.7, 0.8, 0.9	0.91, 0.95, 0.97, 0.99	None
	8	0.2, 0.30, 0.50, 0.78	0.5, 0.65, 0.85, 1.0	0.6, 0.65, 0.85, 0.9	0.91, 0.94, 0.98, 0.99	None
	9	0.2, 0.30, 0.50, 0.78	0.5, 0.65, 0.85, 1.0	0.6, 0.62, 0.82, 0.9	0.91, 0.92, 0.98, 0.99	None
	10	0.2, 0.24, 0.53, 0.78	0.5, 0.6, 0.9, 1.0	0.6, 0.7, 0.8, 0.9	0.91, 0.95, 0.97, 0.99	None
	11	0.2, 0.24, 0.53, 0.78	0.5, 0.6, 0.9, 1.0	0.6, 0.65, 0.85, 0.9	0.91, 0.94, 0.98, 0.99	None
	12	0.2, 0.24, 0.53, 0.78	0.5, 0.6, 0.9, 1.0	0.6, 0.62, 0.82, 0.9	0.91, 0.92, 0.98, 0.99	None
	13	0.2, 0.34, 0.43, 0.78	0.5, 0.7, 0.8, 1.0	0.6, 0.7, 0.8, 0.9	0.91, 0.95, 0.97, 0.99	Standard
	14	0.2, 0.34, 0.43, 0.78	0.5, 0.7, 0.8, 1.0	0.6, 0.65, 0.85, 0.9	0.91, 0.94, 0.98, 0.99	Standard
	15	0.2, 0.34, 0.43, 0.78	0.5, 0.7, 0.8, 1.0	0.6, 0.62, 0.82, 0.9	0.91, 0.92, 0.98, 0.99	Standard
	16	0.2, 0.30, 0.50, 0.78	0.5, 0.65, 0.85, 1.0	0.6, 0.7, 0.8, 0.9	0.91, 0.95, 0.97, 0.99	Standard
	17	0.2, 0.30, 0.50, 0.78	0.5, 0.65, 0.85, 1.0	0.6, 0.65, 0.85, 0.9	0.91, 0.94, 0.98, 0.99	Standard
	18	0.2, 0.30, 0.50, 0.78	0.5, 0.65, 0.85, 1.0	0.6, 0.62, 0.82, 0.9	0.91, 0.92, 0.98, 0.99	Standard
	19	0.2, 0.24, 0.53, 0.78	0.5, 0.6, 0.9, 1.0	0.6, 0.7, 0.8, 0.9	0.91, 0.95, 0.97, 0.99	Standard
	20	0.2, 0.24, 0.53, 0.78	0.5, 0.6, 0.9, 1.0	0.6, 0.65, 0.85, 0.9	0.91, 0.94, 0.98, 0.99	Standard
	21	0.2, 0.24, 0.53, 0.78	0.5, 0.6, 0.9, 1.0	0.6, 0.62, 0.82, 0.9	0.91, 0.92, 0.98, 0.99	Standard

^a^Trapezoidal distribution (minimum value, mode 1 value, mode 2 value, maximum value)

W_1_: Selection probability for abortion-related deaths

W_2_: Selection probability for non-abortion-related deaths

W_3_: Sensitivity of cause of death classification

W_4_: Specificity of cause of death classification

RE: Random Error

### Bias analysis results for study a

Table 2 presents the multiple-bias analysis results for Study A. Study A reported a median of 0.206 (20.6 % of maternal deaths were abortion-related). After adjustment for selection bias under three distribution scenarios, the median increased, on average, to 0.370. After additional adjustment for misclassification, the median proportion of abortion-related deaths increased from the original, on average, to 0.306. After including random error into the multiple-bias analysis, the median was, on average, 0.308, approximately 20 % greater than the reported proportion of abortion-related deaths. Had the authors of Study A reported a 95 % confidence interval around their reported median, it would have been 0.196-0.316. After adjustment for selection bias under three scenarios, the potential range widened to 0.242-0.550. After adjustment for selection bias and misclassification under nine scenarios, the potential range was 0.203-0.458. After including random error in the multiple-bias analysis of selection bias and misclassification, the potential range widened further to 0.169-0.485.

**Table 2:** Multiple-bias analysis results for Study A: proportion of maternal deaths due to unsafe abortion adjusted for selection bias, misclassification, and random error, after 50,000 simulation trials per scenario

Bias model	Scenario (probability distribution/s)	Median	2.5, 97.5 percentiles	Ratio of limits
None (reported)	NA	0.206	0.206, 0.206	1.00
None (conventional, with estimate of precision)	NA	0.206	0.196, 0.316	1.61
Adjusted for misclassification only, no random error	1 (W_1_&W_2_ narrow)	0.369	0.248, 0.523	2.11
	2 (W_1_&W_2_ medium)	0.368	0.245, 0.530	2.16
	3 (W_1_&W_2_ wide)	0.372	0.242, 0.550	2.27
Adjusted for misclassification and selection bias, no random error	4 (W_1_&W_2_ narrow, W_3_&W_4_ narrow)	0.305	0.209, 0.431	2.06
	5 (W_1_&W_2_ narrow, W_3_&W_4_ medium)	0.308	0.210, 0.438	2.08
	6 (W_1_&W_2_ narrow, W_3_&W_4_ wide)	0.302	0.206, 0.432	2.10
	7 (W_1_&W_2_ medium, W_3_&W_4_ narrow)	0.305	0.207, 0.437	2.11
	8 (W_1_&W_2_ medium, W_3_&W_4_ medium)	0.306	0.207, 0.443	2.14
	9 (W_1_&W_2_ medium, W_3_&W_4_ wide)	0.302	0.204, 0.437	2.11
	10 (W_1_&W_2_ wide, W_3_&W_4_ narrow)	0.308	0.206, 0.452	2.19
	11 (W_1_&W_2_ wide, W_3_&W_4_ medium)	0.310	0.206, 0.458	2.22
	12 (W_1_&W_2_ wide, W_3_&W_4_ wide)	0.305	0.203, 0.452	1.96
Adjusted for misclassification and selection bias, random error included	13 (W_1_&W_2_ narrow, W_3_&W_4_ narrow)	0.307	0.175, 0.462	2.64
	14 (W_1_&W_2_ narrow, W_3_&W_4_ medium)	0.310	0.176, 0.469	2.66
	15 (W_1_&W_2_ narrow, W_3_&W_4_ wide)	0.304	0.173, 0.461	2.66
	16 (W_1_&W_2_ medium, W_3_&W_4_ narrow)	0.307	0.174, 0.467	2.68
	17 (W_1_&W_2_ medium, W_3_&W_4_ medium)	0.309	0.173, 0.473	2.78
	18 (W_1_&W_2_ medium, W_3_&W_4_ wide)	0.304	0.170, 0.466	2.74
	19 (W_1_&W_2_ wide, W_3_&W_4_ narrow)	0.311	0.173, 0.480	2.77
	20 (W_1_&W_2_ wide, W_3_&W_4_ medium)	0.313	0.172, 0.485	2.81
	21 (W_1_&W_2_ wide, W_3_&W_4_ wide)	0.308	0.169, 0.480	2.84

W_1_: Selection probability for abortion-related deaths

W_2_: Selection probability for non-abortion-related deaths

W_3_: Sensitivity of cause of death classification

W_4_: Specificity of cause of death classification

Under all 21 scenarios of multiple-bias analysis, our findings show that the increased median proportion of abortion-related deaths provides quantitative evidence that systematic error, specifically selection bias and misclassification, may indeed result in estimates of the proportion of abortion-related maternal deaths that underestimate the true proportion of abortion-related maternal deaths. For Study A, which initially found less than 20 % of maternal deaths to be abortion-related, after multiple-bias adjustment, the proportion of abortion-related mortality was, on average, closer to 30 %.

## Discussion

Multiple-bias analysis provides authors with a set of statistical tools to estimate the influence of biases across a range of plausible magnitudes on the parameters estimated from the study data. In circumstances where systematic error is known to be present, some form of bias analysis should not only be considered a necessary analytic step, but also as a useful framework to help consumers of the literature interpret results vis-á-vis the magnitude and likelihood of potential biases. With some notable exceptions [24, 25, 42], when authors report the results of epidemiologic analyses, they typically do not attempt to quantify the role of bias in those results, even through simple sensitivity analyses, implicitly making the assumption that biases do not exist or are unlikely to change their results. Multiple-bias analysis allows us to exchange those implicit assumptions for explicit assumptions through the quantification of selection bias and sensitivity/specificity. Multiple-bias analysis is particularly applicable to the field of global reproductive health where issues of selection factors, willingness to participate in studies, misreporting, and underreporting of sensitive behaviors have long been acknowledged as obstacles to the collection of high-quality data.

Our finding that the range of possible values for the proportion of abortion-related deaths substantially increased with multiple-bias analysis is further evidence that the current estimates of abortion-related mortality lack both precision and validity. For those interested in quantifying the proportion of abortion-related deaths in any setting, this finding serves as a reminder that given the limitations of our data, we should report not only the observed proportion of abortion-related deaths but also their appropriate confidence intervals and, at the very least, statements about potential sources of bias.

## Limitations

Despite their advantages, multiple-bias analysis techniques have their limitations. Some argue that the results of such analyses are themselves biased by the values chosen for each bias parameter [43]. There is no disputing that the parameters chosen, by their nature, dictate the results of bias analyses. However, by virtue of making a priori statements of the presumed biases and their possible magnitudes (or distributions of magnitudes) in a study, a clear and transparent process by which systematic error was assessed can be established and evaluated by readers [25], who can make their own judgments about the correctness of the authors’ bias parameters.

## Conclusions

Findings from multiple-bias analyses of abortion-related mortality have broad reaching implications for the way we understand the distribution of cause of maternal death in a range of scenarios. If, as our worked example suggests, abortion-related deaths account for a larger proportion of maternal deaths than reported by the study, these methods could be used to more accurately estimate a potential range of abortion-related mortality in local- and country-specific contexts. Such data might also be useful for policymakers and program planners aiming to target funds towards increasing access to legal, safe abortion services at the community level. Such policies and programs will be fundamental to addressing the issue of mortality resulting from unsafe abortion and achieving the SDGs.

With some fairly simple steps, reporting results of multiple-bias analyses in estimates of abortion-related mortality, predictors of unsafe abortion, and other reproductive health questions that suffer from similar biases, would improve reporting practices in the field, in addition to the possibility of providing a more accurate understanding of the range of potential impact of policies and programs that target the underlying causes of unsafe abortion and abortion-related mortality.

## Additional file

Additional file 1: R Code for Analysis. (DOCX 15 kb)
