# Supplementary material for: Multiple-bias analysis as a technique to address systematic error in measures of abortion-related mortality
Source: Popul Health Metr. 2016 Mar 22;14:9. doi: 10.1186/s12963-016-0075-3 (PMC4802921; doi:10.1186/s12963-016-0075-3)
Supplement: Additional file 1: — R Code for Analysis. (DOCX 15 kb) [file 12963_2016_75_MOESM1_ESM.docx]

**R Code for Analysis**

library(trapezoid)

n=50000

number_ARD=52

number_NARD=253

#W1= selection probability for ARD

W1=rtrapezoid(n,min=0.02, mode1=0.34, mode2=0.43, max=0.78, n1=2, n3=2, alpha=1)

#W2 = selection probability for non-ARD

W2=rtrapezoid(n,min=0.5, mode1=0.7, mode2=0.8, max=1, n1=2, n3=2, alpha=1)

#W3 = sensitivity of cause of death classification

W3=rtrapezoid(n,min=0.6, mode1=0.7, mode2=0.8, max=0.9, n1=2, n3=2, alpha=1)

#W4 = specificity of cause of death classification

W4=rtrapezoid(n,min=0.91, mode1=0.95, mode2=0.97, max=0.99, n1=2, n3=2, alpha=1)

W1

W2

W3

W4

#Y0=rep(number_ARD/number_NARD,n)

pbar_0=(52/253)

SE_0=sqrt(pbar_0*(1-pbar_0)/(128))

SE_0

E_0=qnorm(.975)*SE_0; E_0

pbar_0+c(-E_0,E_0)

Y0=pbar_0+c(-E_0,E_0)

X_1ARD=(number_ARD/W1)

X_1NARD=(number_NARD/W2)

#Y1

Y1=(X_1ARD/(X_1NARD+X_1ARD))

X_2ARD=((X_1ARD*W3)+(X_1NARD-(X_1NARD*W4)))

X_2NARD=((X_1NARD*W4)+(X_1ARD-(X_1ARD*W3)))

#Y2

Y2=(X_2ARD/(X_2NARD+X_2ARD))

#Y3

pbar=Y2

SE=sqrt(pbar*(1-pbar)/(X_2NARD+X_2ARD))

SE

E=qnorm(.975)*SE; E

pbar+c(-E,E)

Y3=pbar+c(-E,E)

Y0

Y1

Y2

Y3

X_1ARD

X_1NARD

X_2ARD

X_2NARD

#Y0

rep (mean (Y0), n)

x<- seq(0,253,by=1)

y<-dbinom(x, 253,.2055)

plot (x,y)

quantile(Y0, probs=c(0.025,0.5,0.975))

hist(Y0, breaks=50, col="lightblue", border="gray", xlab="Proportion of Abortion related deaths with random error")

#Y1

quantile(Y1, probs=c(0.025,0.5,0.975))

hist(Y1, breaks=100, col="lightblue", border="gray", plot=TRUE, xlim=c(0, .3), xlab="Selection Bias Adjusted ARD")

#Y2

quantile(Y2, probs=c(0.025,0.5,0.975))

hist(Y2, breaks=100, col="lightblue", border="gray", plot=TRUE, xlim=c(0, .3), xlab="Misclassification AND Selection Bias Adjusted ARD")

#Y3

quantile(Y3, probs=c(0.025,0.5,0.975))

hist(Y3, breaks=100, col="lightblue", border="gray", plot=TRUE, xlim=c(0, .3), xlab="Misclassification and Selection Bias Adjusted ARD with Random Error")
